# Thermo-Mechanical Behaviour of Flax-Fibre Reinforced Epoxy Laminates for Industrial Applications

**DOI:** 10.3390/ma8115384

**Published:** 2015-11-03

**Authors:** Giuseppe Pitarresi, Davide Tumino, Antonio Mancuso

**Affiliations:** 1 Dip. di Ingegneria Chimica, Università degli Studi di Palermo, Gestionale, Informatica, Meccanica (DICGIM), Palermo 90128, Italy; giuseppe.pitarresi@unipa.it (G.P.); antonio.mancuso@unipa.it (A.M.); 2 Facoltà di Ingegneria e Architettura, Università degli Studi di Enna “Kore”, Enna 94100, Italy

**Keywords:** flax fibre composite, tensile properties, crimped unidirectional textiles, damage, IR thermography, thermoelastic stress analysis

## Abstract

The present work describes the experimental mechanical characterisation of a natural flax fibre reinforced epoxy polymer composite. A commercial plain woven quasi-unidirectional flax fabric with spun-twisted yarns is employed in particular, as well as unidirectional composite panels manufactured with three techniques: hand-lay-up, vacuum bagging and resin infusion. The stiffness and strength behaviours are investigated under both monotonic and low-cycle fatigue loadings. The analysed material has, in particular, shown a typical bilinear behaviour under pure traction, with a knee yield point occurring at a rather low stress value, after which the material tensile stiffness is significantly reduced. In the present work, such a mechanism is investigated by a phenomenological approach, performing periodical loading/unloading cycles, and repeating tensile tests on previously “yielded” samples to assess the evolution of stiffness behaviour. Infrared thermography is also employed to measure the temperature of specimens during monotonic and cyclic loading. In the first case, the thermal signal is monitored to correlate departures from the thermoelastic behaviour with the onset of energy loss mechanisms. In the case of cyclic loading, the thermoelastic signal and the second harmonic component are both determined in order to investigate the extent of elastic behaviour of the material.

## 1. Introduction

The development of eco-friendly composite concepts by employing raw materials from natural renewable sources is a strong driver in actual research on lightweight structures [1]. Since a few years, a number of natural fibre reinforcements, in the form of fabric textiles or pre-preg assemblies, have made their way as commercial products, readily available to the composite industry for direct employment in traditional manufacturing processes as well. Even so, the use of such materials is still somewhat inhibited by a lack of knowledge or predictability of the mechanical behaviour of the bulk composite, especially with reference to accumulation of damage or other irreversible processes under both static and cyclic loading [2].

Flax fibres are actually the leading choice among natural bast fibres, for their slightly superior structural performances, but also for their comparatively more competitive costs and weight [1,3,4,5]. The assembly of natural flax fibres into continuous reinforcements is essential for achieving adequate stiffness and strength for structural applications [6]. The actual most common type of such long reinforcement consists of yarns of twisted fibres, usually assembled into woven fabrics or, in a few cases, even pre-preg tapes. The helical structure resulting from yarn spinning provides only a quasi-unidirectional alignment of elementary fibres, with the twisting angle usually ranging between 5 and 20 degrees [4,7]. If such twisting angle is too low, the dry yarn might end up having poor tensile strength, hampering its processability [6]. A high twist angle would instead results in an excessive off-axis orientation reducing the mechanical performances of the final composite [6,7].

**Figure 1:**
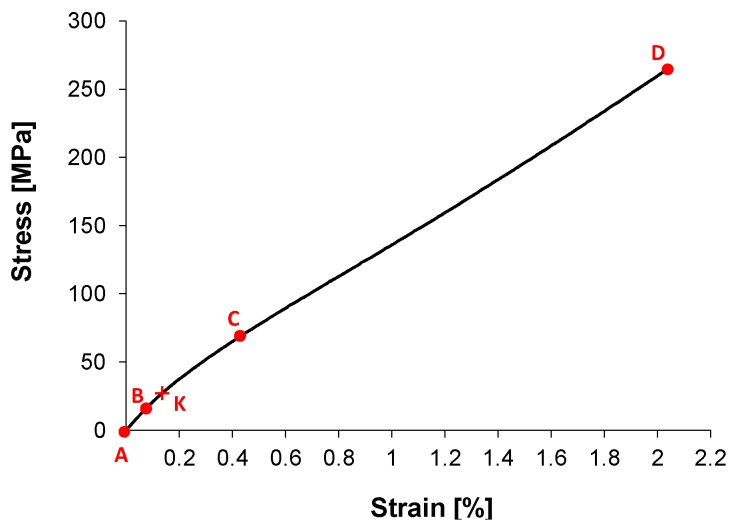
Typical tensile curve of a unidirectional (UD) Flax Fibre Reinforced Composite (FFRC) along the reinforcement orientation.

In order to assess the final macroscopic reinforcing effect, tensile samples from unidirectional (UD) composites are then primarily tested along the reinforcement direction. Data in the literature from flax, but also from other bast fibre reinforced UD composites, have shown a peculiar behaviour, exemplified by the stress-strain curve shown in Figure 1 [4,8,9,10,11,12,13,14,15,16,17,18]. A bilinear trend is observed, with a first linear elastic region AB, followed by a nonlinear interval BC and a successive quasilinear stage CD up to brittle failure. The change of slope between AB and CD determines a rather marked change of stiffness, generally in the order of 30%–50%. The first linear stage AB ends at relatively low stress and strain levels (in the order of 0.1%–0.3%). The final quasilinear CD stage sometimes exhibits a slightly increasing (hardening) or decreasing (softening) trend. Such bilinear tensile behaviour for UD composites is found almost unchanged for both thermoplastic [12,13] and thermosetting matrix composites, as well as for twisted yarns, in prepregs and dry fabrics, low twisted rovings and also untwisted and non-crimped assemblies [4,14]. In light of this, the behaviour is most likely to be correlated with the intrinsic strain behaviour of elementary fibres [19].

The mentioned marked and early reduction of composite stiffness should raise some big concerns among designers and final users, but actually only a few works yet have fully addressed such macroscopic features of plant continuous fibre reinforced composites.

In particular, some works explain the change of slope in the tensile curve as the macroscopic manifestation of a collective viscoelastic reorientation of Micro-Fibrils present in the secondary cell walls of each elementary flax fibre [9,15,16,19]. Shah *et al.* [9] also suggest that a contribution to the non-linear behaviour might arise from the untwisting/stretching of the fibre yarns, although the tensile non linearity has been observed also with un-twisted fibre assemblies [4,8,10]. A few works [8,14,16] have found out that in the knee region of the tensile curve (BC in Figure 1) only low Acoustic Emission events are detected, which might indicate that changes are induced by silent Micro-Fibrils reorientation or intra-fibre microstructural failures rather than noisier crack damage events at the mesoscale. In particular, Kersani *et al.* [14] suggest that the non-linearity of the composite and of the single fibres under tension are directly correlated, and reported that fibre/matrix debonding failures start at strain values only twice those of the yield knee (point K in Figure 1). It is widely accepted that the progressive alignment of Micro-Fibrils along the load direction is the cause for the late increase in stiffness in elementary fibres [9,18,19]. Such increase, though, does not seem to fully transfer in the UD composite (CD range of Figure 1), where a quasi-linear trend is observed up to brittle failure. Kersani *et al.* [14] suggest that this lack of stiffness improvement is due to the fabric crimp and yarn twists, which determine a further misorientation of elementary fibres, simultaneously hampering the elementary fibres reaching the hardening stage. In this regard, it is observed that a few works in the literature, testing UD composites employing non-crimped and un-twisted fibre assemblies, indeed report some evidence of hardening in the CD stage of the tensile curve, which might be a sign of a higher impact of Micro-Fibrils’ reorientation for such straight reinforcements [4,8,10,15].

Charlet *et al.* [15] suggest that the non-linear tensile behaviour of composites is also correlated to internal sliding between bundled fibres, *i.e.*, agglomerated fibres which have not been properly separated in the fibres’ extraction process [18]. In fact, within such bundles, the matrix resin has not penetrated, and fibres are bound together by a weak pectin interface. Also, Ramoney *et al.* [20] identify early evidence of fibre–fibre interface debonding by using Acoustic Emission techniques.

Some authors have also correlated the non-linear tensile behaviour of elementary fibres to the influence of local kink bands and dislocation defects [19,21]. Kink bands are usually introduced by the isolation process of fibres from the plant, and determine local low stiffness bulged regions, which interrupt the geometrical and mechanical continuity of the fibre. After a certain load, threshold kink bands tend to straighten up, thus contributing to the temporary non-linear load increase and stiffness reduction. A further interesting finding by Bailey [19] is that, by repeating loading-unloading cycles, the tensile curve evolves towards a more linear Hookean behaviour.

Hughes [8] suggests that kink band defects have also a fundamental role in determining the non-linear behaviour of UD composites. Under tensile loading, the low stiff kink bands produce local stress concentrations in the matrix, leading to early local fibre/matrix debonding, which Hughes also links to low-level acoustic emission detected at the onset of the non-linearity. Further evidence is found by Hughes [8] by reporting that an improved fibre/matrix shear strength, achieved by special fibre sizing, determines a higher yield point and a subsequent smaller stiffness reduction.

While the majority of quoted studies have investigated monotonic tensile behaviours, some more recent works have extended the analysis by applying low speed repeated loading/unloading cycles [9,10]. These works have highlighted some further interesting features: cycling with load amplitudes higher than the yield point (K in Figure 1) gives rise to a permanent inelastic deformation and to a certain amount of energy loss (area of hysteresis of cycles); the major amount of energy loss is comprised in the first cycles; immediate reloading after previous cycling finds the material stiffness and yield point substantially modified from the values of the virgin material. Such findings demonstrate the onset of some levels of irreversible modifications, activated by loading above the knee yield point, whose knowledge, still very limited, is though fundamental for a correct modelling of the material at the design stage.

This work presents results of tensile tests on UD composites made from a quasi-unidirectional flax fabric using twisted yarns. Three lamination procedures have been implemented giving rise to different fibre volume fractions. Two characterizations are in particular proposed. In the first one, the stiffness response of the material is investigated after repeated monotonic and cyclic loadings, with load cycles having different ratios of *σ_min_*/*σ_max_* (*R*-ratio). In the second, Infrared Thermography is used to monitor the full field temperature of specimen during monotonic and cyclic loading. In the case of monotonic loading, the thermal signal is analysed to correlate departures from the linear thermoelastic behaviour with the onset of energy loss mechanisms. The thermal signal acquired during cycling is instead post-processed to evaluate the Thermoelastic Signal. Maps of the Thermoelastic signal component and the second harmonic component are in particular obtained for cycles with different *R*-ratios, allowing to investigate the extent of elastic behaviour of the material.

## 2. Materials and Processes

### 2.1. Raw Materials

**Figure 2:**
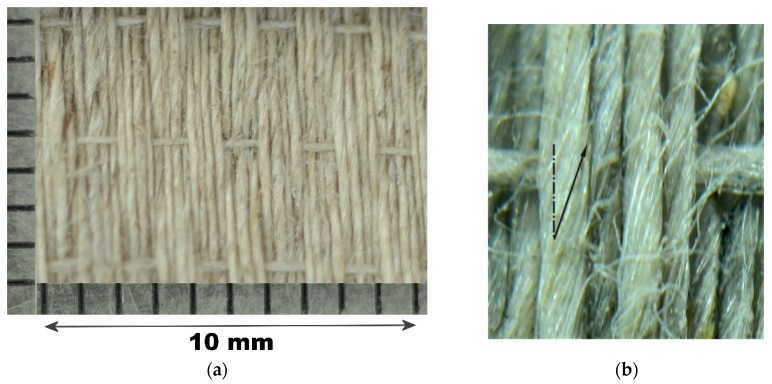
(**a**) Macro photo of the fabric; (**b**) Close-up photo revealing the twisted fibres in yarn filaments.

The flax reinforcement used in this work is a quasi-unidirectional woven flax fabric Flaxdry UD-180-C003 supplied by *Lineo nv* (Meulebeke, Belgium). The fabric is a regular plain weave 4/4 weft rib structure (see Figure 2a), with 42.5 ends/cm in the warp direction and 3 picks/cm in the weft. Weft yarns have a lighter linear density (27.8 Tex) than warp yarns (41.7 Tex), and the overall nominal areal density is 190 g/m^2^. Yarns are made by Z twisted fibres, with 420 twists/m in the warp and 520 twists/m in the weft direction (see also Figure 2b).

Two commercial epoxy resins have been used: MATES SX-10 EVO (with a Medium hardener) for hand lay-up impregnation, and MATES SX-8 EVO for resin infusion impregnation, both supplied by *Mates srl* (Segrate, Italy). Both resin systems achieved a full cure at room temperature, allowing one week resting before samples cutting.

### 2.2. Laminate Fabrication and Plan of Experiments

Three UD panels with lay-up [0°]_6_ have been manufactured, each using a different technique: Hand Lay-Up, Vacuum Bagging and Resin Infusion (hereinafter indicated as HL, VB and RI). The HL and VB processes both use hand lay-up impregnation, but the VB panel is vacuum bagged after impregnation and excess resin extracted during curing, while the HL panel is placed between two glass plates and put under a light pressure to ensure only good dimensional stability and uniform thickness. The RI panel employed a classic vacuum resin infusion process, with the dry lay-up assembly vacuum bagged and then impregnated by vacuum driven resin flow. Further details of the manufactures are given in two companion papers of this work [22,23], which investigate the flexural behaviour of sandwiches made with a cork agglomerated foam core and flax skins of the same material analysed here.

The three manufactured monolithic panels had dimensions of 350 mm × 350 mm. This allowed obtaining about 11 longitudinal 250 mm × 15 mm [0°]_6_ and five transverse 175 mm × 25 mm [90°]_6_ samples for tensile testing according to ASTM D 3039M, and also a residual rectangular area which was useful to obtain a single three rail shear sample according to ASTM D 4255, for the evaluation of in-plane shear rigidity.

Direct measurement of the volume and weight of the composite, the density of the cured polymer matrix, and the areal weight of the fabric, allows the evaluation of the fibre volume fraction *v_f_* for the three panels (see also [8,12,22]), reported in Table 1. This estimation is only ideal as it assumes a zero void content, thus overestimating *v_f_*. A probably more realistic estimation can be done based on the knowledge of the flax fibres density, by means of the following equation [9,12]:

(1) $$v_{f}=\frac{m_{f}}{\rho_{f}}\frac{1}{V_{c}}$$

where *m_f_* is the total fibres mass weight, obtained from the fabric areal weight, *ν_f_* is the flax fibres density and *V_c_* is the volume of the composite sample used for the calculation. Results of *v_f_* calculated from Equation (1), based on the assumption that *ν_f_* = 1.4 g/cm^3^ [12,24], are also reported in Table 1 for comparison.

**Table 1:** Estimation of Fibres Volume Fractions *v_f_* (%).

Procedure	HL	VB	RI
Neglecting void content	39 ± 1	51 ± 3	42 ± 1
Assuming flax fibres density (Equation (1))	30 ± 0.4	38 ± 0.4	36 ± 0.7

## 3. Mechanical Characterisation: Results and Discussion

Tensile tests on longitudinal samples have been performed with three different loading conditions: quasi-static monotonic, quasi-static loading-unloading, and fatigue cycling (at 4 Hz). These tests were performed on a servo-hydraulic 100 kN MTS 800 testing machine, equipped with hydraulic wedge grips and digitally controlled by MTS FlexTest SE. The deformation sampling was synchronised to load sampling by connecting and conditioning a two-sided HBM DD1 extensometer with the same FlexTest SE controller. It is worth reporting that tensile tests were repeated with three extensometer gauge lengths, *i.e.*, 25, 50 and 100 mm.

### 3.1. Quasi-Static Monotonic Tests

Monotonic tensile loading tests were performed in displacement control, at a constant crosshead speed of 2 mm/min, which provided axial strain rates close to 0.01 min^−1^ (see also Section 3.2 for a comparison with load controlled quasi-static tests). Table 2 summarises the findings of the standard characterisation performed according to ASTM D3039. The Transverse modulus is in particular measured on [90°]_6_ samples loaded by a electro mechanic Instron 3367 machine equipped with a 1 kN load cell. The Poisson ratio was obtained by measuring the transverse strain from an electrical strain gauge bonded in the area between the knives of the extensometer, with the two strain devices conditioned and synchronised by an external Wheatstone bridge data logger.

**Table 2:** Stiffness and strength measured parameters (*E* Young’s modulus, *G_LT_* shear stiffness, *σ_K_* strain at knee yielding, *σ_U_* ultimate brittle failure stress, *σ_U_* strain at failure).

Fabrication Process	*V_f_* (%)	*E_L_* (GPa)	*E_T_* (GPa)	*ν_LT_*	*G_LT_* (GPa)	*σ_K_* (MPa)	*σ_K_* (%)	*σ_U_* (MPa)	*σ_U_* (%)
HL	39	17.7 ± 0.55	3.6 ± 0.27	0.345	1.33	30 ± 3.6	0.16	238 ± 15.7	2.11
VB	51	19.0 ± 0.67	3.0 ± 0.24	0.401	1.33	36 ± 1.7	0.16	282 ± 20.8	2.11
RI	42	21.7 ± 0.49	3.8 ± 0.10	0.330	1.57	33 ± 2.7	0.16	267 ± 3.5	2.07

**Figure 3:**
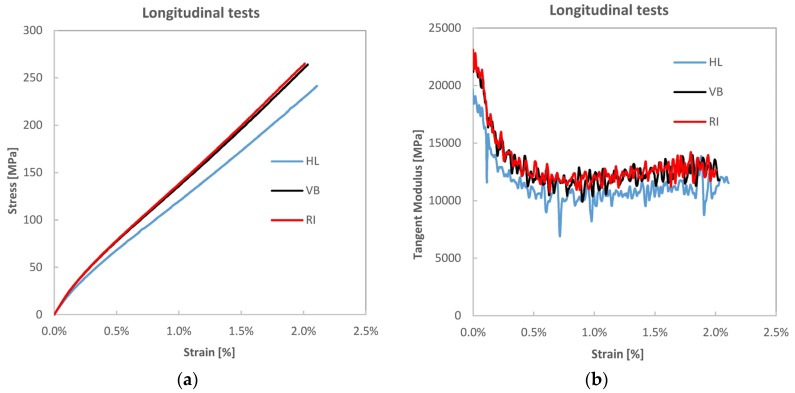
(**a**) Tensile stress *vs.* strain curves for the three manufactured batch types and (**b**) tangent modulus *vs.* strain.

Figure 3a shows an example of tensile curves in the longitudinal loading direction for each process type. Young’s modules in Table 2 are tangent modules at the initial linear elastic region of the curve. Figure 3b shows the evolution of the tangent modulus with strain. A representative post-elastic modulus is calculated by linear fitting of the CD range of the tensile curve (see Figure 1 and Figure 3a). This is reported in Table 3 and identified by the subscript PK which stays for the post knee region, for direct comparison with the original tangent modulus identified by the subscript BK (before the knee region).

**Table 3:** Comparison of Young’s modules in the quasilinear regions before and after the knee.

Fabrication Process	*E_LBK_* (GPa)	*E_LPK_* (GPa)	*E_LBK_* (GPa)	*E_LPK_* (GPa)	*E_LBK_* (GPa)	*E_LPK_* (GPa)
	*First Monotonic Test*		*Second Monotonic Test*		*Third Monotonic Test*	
HL	17.7	9.7	18.7	11.7	18.4	11.5
VB	19.0	12.3	21.0	14.2	22.3	14.2
RI	21.7	12.0	22.8	14.9	22.7	15.1

It is observed that all three composites obtained with the three lamination processes described in Section 2.2 have exhibited the peculiar nonlinear behaviour sketched in Figure 1 and widely reported in the literature for all similar composites. Young’s modules and strengths in the yarn’s direction are increasing with the fibre volume fraction, although not necessarily as predicted by rule of mixture models. It is also reported that the measured values of stiffness did not show any meaningful change correlated with the extensometer gauge lengths (*i.e.*, 25, 50, 100 mm), which allows stating that the non-linear evolution of the tensile curve is an intrinsic property of the material. For all laminates, a rather steep stiffness drop is observed (see Figure 3b and Table 3) between 35% and 45%, with values of stress and strain at the knee yield point similar for all three materials (Table 2). In particular, for all materials the stress at the knee point amounted to 12%–13% of the ultimate tensile stress, while strain at the knee point occurred at about 8% of the ultimate strain. It is observed that the longitudinal modulus has a slightly increasing trend in the CD region of the tensile curve, which could be related to a reduction of the twisting angle of yarns with tensile load.

**Figure 4:**
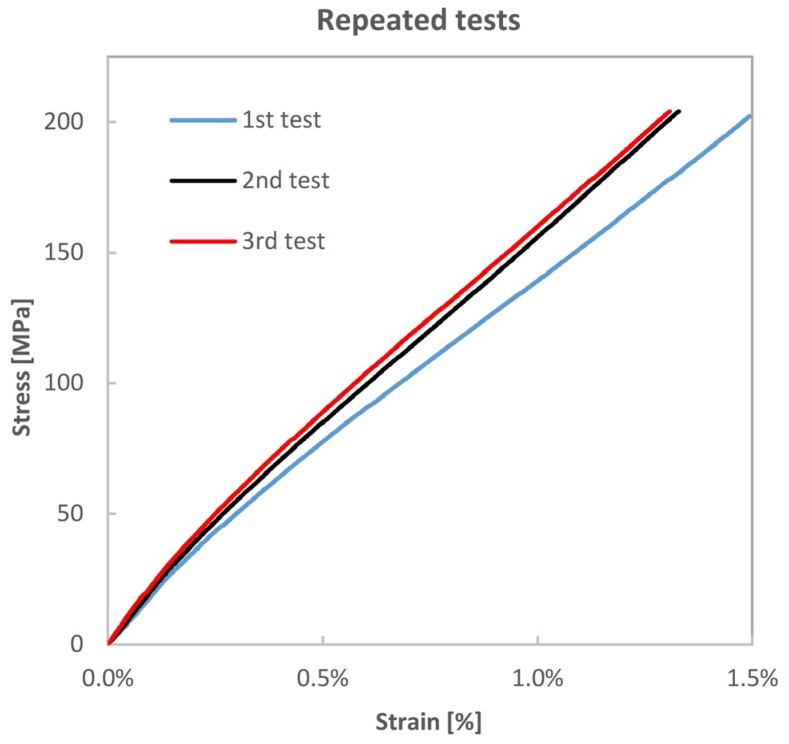
Evolution of the tensile curve for the same VB specimen retested after 24 h for three times.

A number of tensile samples from each batch were loaded in tension three times, at intervals of about 24 h, taking care to stop the load little before the onset of ultimate brittle failure (about 80% of *σ_U_*). Figure 4 compares the three measured tensile curves for a VB sample. The observed behaviour is similar for all three batches of materials, and shows a progressive stiffness hardening, more marked between the first and the second loadings, and tending to stabilise after the third loading. The values of Young’s modules for the repeated tensile loadings are also reported in Table 3.

**Figure 5:**
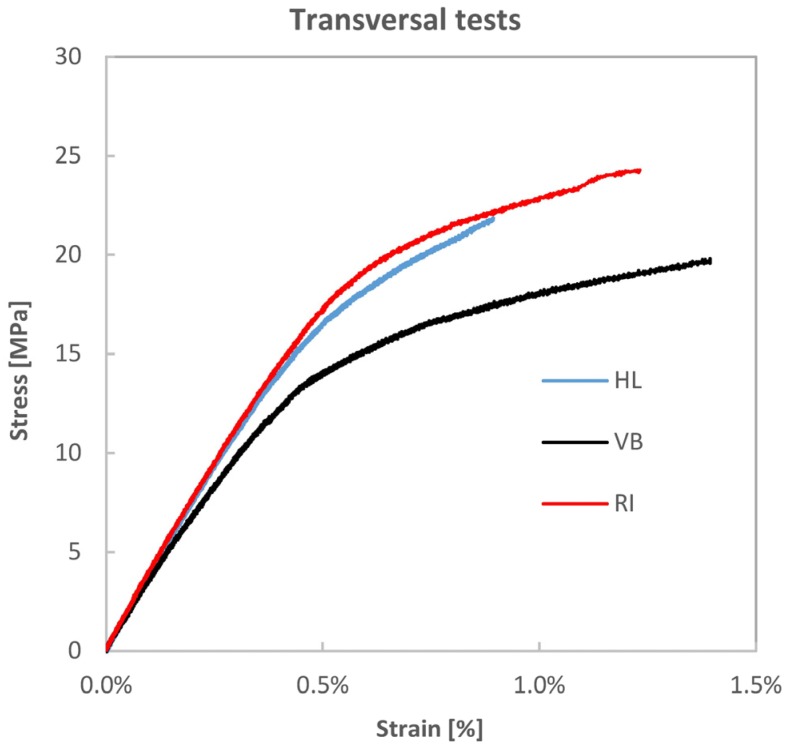
Transverse tensile stress *vs.* strain curves.

Figure 5 shows the measured tensile stress *vs.* strain up to failure in the transverse direction, used for the evaluation of the transverse modulus reported in Table 2. Fibre volume fraction seems to have a detrimental influence on stiffness properties related with the material off-axis behaviour, *i.e.*, the transverse modulus *E_T_* and Poisson’s ratio *ν_LT_* (the value of *ν_LT_* reported in Table 2 is referred to the initial linear elastic region). In fact, the VB composite exhibits the smaller *E_T_* and bigger *ν_LT_*. This is explained by the intrinsic strong anisotropy and poor off-axis behaviour of flax fibres, which is probably a weakening factor for the matrix resin itself, considering the low values of *E_T_* that have been obtained (see Table 2).

**Figure 6:**
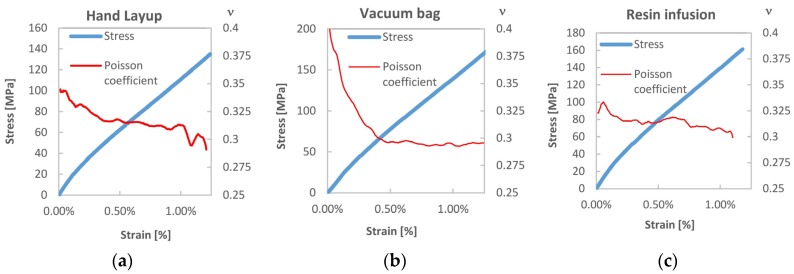
Evolution of Poisson’s coefficient with strain for the: (**a**) hand lay-up; (**b**) vacuum bag and (**c**) resin infusion laminated composites.

Figure 6 reports the evolution of the Poisson’s ratio *ν_LT_* with the progression of deformation in longitudinal tensile samples. It is interesting to observe how this coefficient tends to decrease with loading, and in particular moving from the first to the second pseudo linear regions of the tensile curve. Such decrease, observed in all materials, is particularly marked for the VB sample, *i.e.*, the batch with the highest fibre volume fraction.

The bigger value of *ν*_LT_ at low strains might be related to the presence of bulge kink bands. These probably offer less transverse stiffness initially, which then gradually increases with the straightening up of such bands as load increases. Another explanation could be the effect of looser elementary fibres in the yarns, which may compact with the increase of load, thus developing higher stiffness in the transverse direction.

### 3.2. Quasi-Static Loading-Unloading Tests

**Figure 7:**
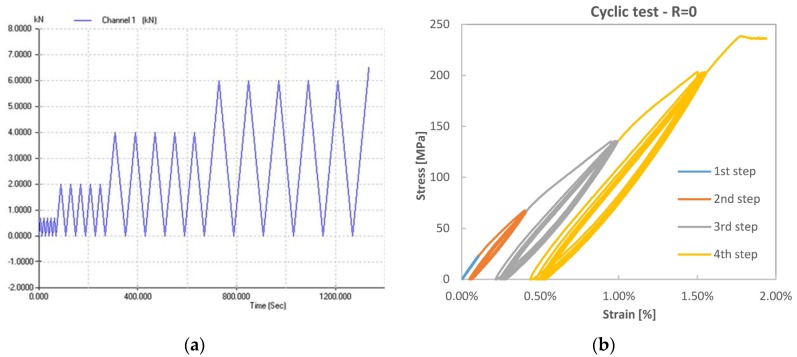
Tensile test with embedded load-unload R = 0 cycles for a VB sample. (**a**) Definition of load-controlled cycling history; (**b**) Stress-strain plot.

The nature of the inelastic behaviour beyond the knee region was investigated by applying quasi-static load-unload cycles. Using the *Multi Purpose Testware* tool of the MTS Series 793™ Control Software, it was possible to apply a load history as shown in Figure 7, consisting of a succession of four groups of five saw-teeth shaped load-unload cycles from zero to a peak load varying for each group: 0.8 kN, 2 kN, 4 kN, 6 kN. The first group has a peak load below the initiation of the non-linear BC region. Although the test is performed in load control, the test speed was selected such to maintain the strain rate close to 0.01 min^−1^, which is also the advised test speed for quasi-static tensile tests according to ASTM D 3039. An example of the obtained tensile curves is shown in Figure 7 for a VB sample. This behaviour was qualitatively similar for all batches.

The comparison of Figure 4 and Figure 7 reveals some remarkable differences between immediate quasi-static loading-unloading cycles and reloading after about one day of recovery time. Reloading after recovery produces a behaviour much similar to that of the virgin material, with a slight stiffness hardening effect limited to the first few loadings. The extent of such hardening is quantitatively described in Table 3 for three loadings. The quasi-static loading-unloading behaviour, reported in Figure 7, seems to confirm the findings of Hughes *et al.* [8] and Newman *et al.* [10], with a rather marked residual strain measured after unloading from stress values above the knee point.

The loading-unloading stress-strain cycles also present an important hysteresis area, which increases with the stress value at which unloading starts, and decreases with the evolution of loading-unloading cycles. As already shown also in [8,9,10], during the loading-unloading cycles, the material exhibits a somewhat increased average stiffness, close to that of the initial region AB. When the load is re-increased to higher values than that previously reached (see Figure 7), the slope of the stress-strain curve comes back to that of the post-knee CD region found in the monotonic test. Retesting after a resting time instead shows that the material recovers the non-linear region and the knee point typical of the virgin material, with just small variations in the new position of the knee point. Such recovery with time provokes the thought that viscoelastic phenomena, more likely occurring within the elementary fibres, have a paramount role.

### 3.3. Fatigue Cycling Tests

A further investigation into the cyclic behaviour of the material was carried out by applying sinusoidal cycles at values of *R*-ratios: 0 < *R* < 1. Each interval of cyclic loading lasted about 2 min, and during such a time window an IR camera was used to sample the specimen temperature for evaluation of the thermoelastic signal (see Section 4). Cycling was carried out at 4 Hz at different values of mean stress and stress amplitude, during a monotone tensile load. The loading frequency value of 4 Hz was in particular chosen as it ensured the onset of adiabatic conditions for the Thermoelastic Stress Analysis (see Section 4). It also allowed the IR camera to acquire a high number of samples at a relatively moderate IR camera frame rate (100 Hz), improving the performances of the lock-in signal filtering for the evaluation of the thermoelastic signal (see Section 4.2).

**Figure 8:**
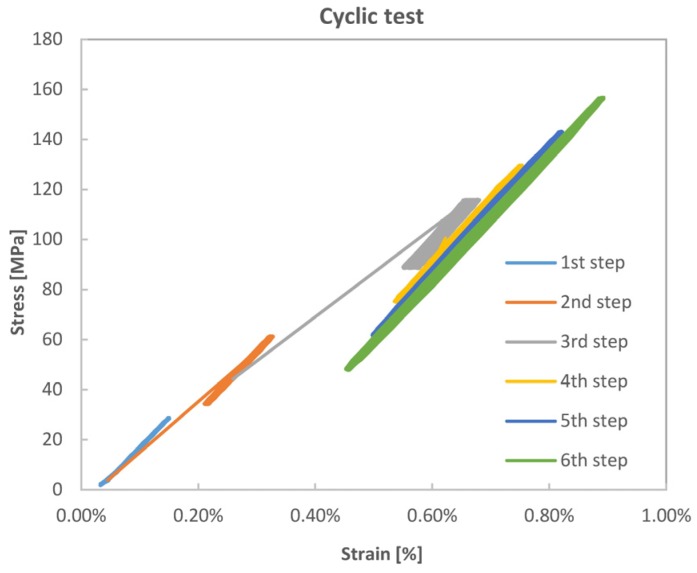
Tensile stress *vs.* strain curve with application of six step cycling loads.

The stress *vs.* strain acquired during the whole test is shown in Figure 8 for a VB sample (also in this case taken as representative of all batches of material). Table 4 shows the mean and amplitude loads defining the six cyclic loading steps. It is in particular observed that the first step was carried out within the AB initial linear elastic range of tensile behaviour. The second step was entirely lying on the post knee CD region, but in the initial part of such region. Steps 3–6 were still taken within the CD region, but with a higher mean load and an increasing load amplitude.

**Table 4:** Values of load steps.

Cycling Loads	Mean Load (N)	Peak to Peak Amplitude (N)
1st step	450	800
2nd step	1400	800
3rd step	3000	800
4th step	3000	1500
5th step	3000	2500
6th step	3000	3000

**Figure 9:**
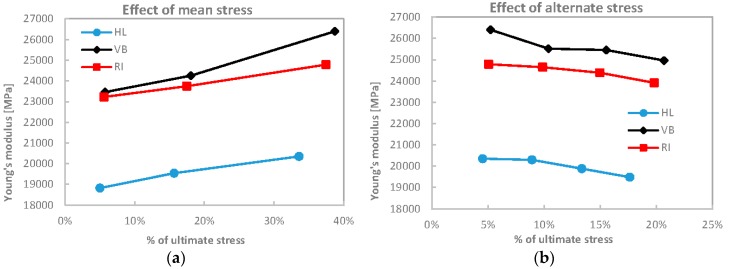
Evolution of Young’s modules with mean (**a**) and amplitude (**b**) stress during cycling loading.

During cycling, the material, in particular, exhibited a linear behaviour and the slope of the stress strain curve, still referred as a Young’s modulus, showed a slight increase with mean load and a decrease with load amplitude as evidenced in Figure 9.

Contrary to the cycles of Figure 7, the cycles shown in Figure 8 are designed as such to not cross the knee point, and to interfere as little as possible with the non-linear BC region. Another difference with Figure 7 lies in the strain rate. Loading-unloading cycles of Figure 7 were quasi-static with a strain rate of about 0.01 min^−1^, while cycles in Figure 8 are applied at a frequency of 4 Hz, with strain rates ranging between 0.48 min^−1^ and 1.8 min^−1^. A first remarkable feature of cycles in Figure 8 is their almost null hysteresis (small only for the 6th step cycle, see Table 4). Furthermore, the slope of the curve during cycling is systematically higher than the slope of the monotonic curve, and increasing with the increase of the average stress (see for instance Figure 9a). This increased stiffness is probably the effect of the more aligned fibre, both in terms of kink bands and Micro-Fibril angles, while the high strain rates do not activate the viscoelastic nonlinear deformation, preserving the stiffness and hampering hysteresis dissipation. A slight decrease of stiffness is instead detected at increasing load amplitudes and constant average load (loading steps 3–6 in Table 4).

## 4. Thermal Analysis

An Infrared (IR) Thermocamera was used to sample the specimen temperature during tensile tests. A cooled sensor high thermal resolution Flir X6540sc model was, in particular, used in combination with the FLIR Research IR 3.4 software for remote control and thermograms basic processing.

The temperature was acquired on two specific occasions:

1. during the monotone quasi-static loadings reported in Figure 3 and Figure 4;
2. during a time window of 30 s within each of the six steps of cyclic loading shown in Figure 8.

If a linear elastic behaviour characterises the material, then the only thermo-mechanical coupling should be represented by the Thermoelastic effect [25]. This means that temperature should follow a variation according to the Thermoelastic law for orthotropic materials, given by [26]:

(2) $$∆T=-T\frac{\alpha_{L}}{\rho C_{p}}(∆\sigma_{L}+\frac{\sigma_{T}}{\sigma_{L}}∆\sigma_{T})$$

where *α* is the Coefficient of Thermal Expansion, *ρ* and *C_p_* density and specific heat at constant pressure of the bulk material, and *T* the absolute mean temperature of the sample. Subscripts *L*,*T* in the tensorial parameters refer to the material principal directions, *i.e.*, the yarns warp direction for *L* and the weft direction for *T*. For unidirectional tensile loading, by indicating with *1* and *2,* respectively, the higher and lower principal stress directions, two scenarios are possible:

- the surfacing fibres are aligned with load direction, *i.e.*, *L* = *1*. In this case, it is *Δσ_L_* > 0 and *σ_T_* = 0 (second term in parenthesis in Equation (2) becomes null);
- the surfacing fibres are transverse to the load, *i.e.*, *T* = *1* and *L* = *2* (this is for instance the case of the surfacing weft ties of the fabric). In this case it is *Δσ_L_* = *Δσ_2_* = 0 and *Δσ_T_* = *Δσ_1_* > 0 (first term in parenthesis in Equation (2) becomes null).

### 4.1. Thermographic Analysis of Monotonic Loading Tests

Assuming *Δ_L_*>0 in Equation (2) (see e.g., [27]), the evolution of sample temperature during monotonic elastic loading should be linearly decreasing. Some authors [28,29] have indicated a departure from linearity and an inversion of trend as a sign that the material is starting to experience some inelastic deformation or damage which are generally associated with the generation of irreversible heating opposing the thermoelastic cooling trend.

**Figure 10:**
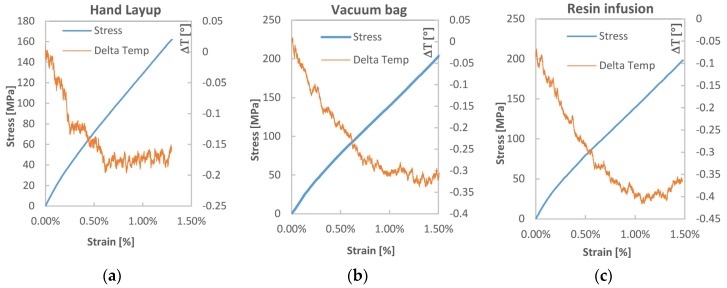
Evolution of temperature during monotonic tensile tests overlapped to the stress strain curves for for the: (**a**) hand lay-up, (**b**) vacuum bag and (**c**) resin infusion laminated composites.

Figure 10 shows the evolution of temperature measured during monotonic tensile tests. This is superpositioned to the stress-strain curves for each batch of material, in order to correlate the departure of temperature from linearity with the level of strain reached by the material. The plotted temperature is the average value over a central area of the sample comprising about 3000 pixels.

Results of the temperature measurements (Figure 10), although quite noisy, do show a quasi-linear initial trend, extending for a temperature decrease of about 0.2 °C. This first trend starts to depart from linearity at values of strain generally above 0.5%, and hence well after the non-linear BC region of the tensile curve. The non-linear behaviour tends to an inversion from temperature decrease to temperature increase, determining a minimum point that is fully onset at values of strain above 1%. If such departure from linearity is to be correlated to dissipative micro-damage setting on the material, it is then meaningful to highlight how such evidence starts only after the second linear region CD of the curve has onset. This occurrence can be linked to the acoustic emission results presented by Kersani *et al.* [14], who also indicated the development of damage related acoustic activity at similar levels of the tensile curve.

### 4.2. Thermoelastic Stress Analysis

**Figure 11:**
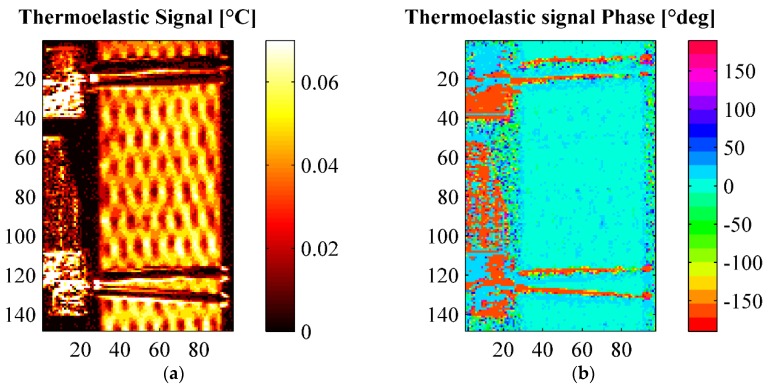
Thermoelastic signal amplitude and phasefrom an HL sample under loading step 1 (see Table 4). (**a**) Amplitude; (**b**) Phase.

**Figure 12:**
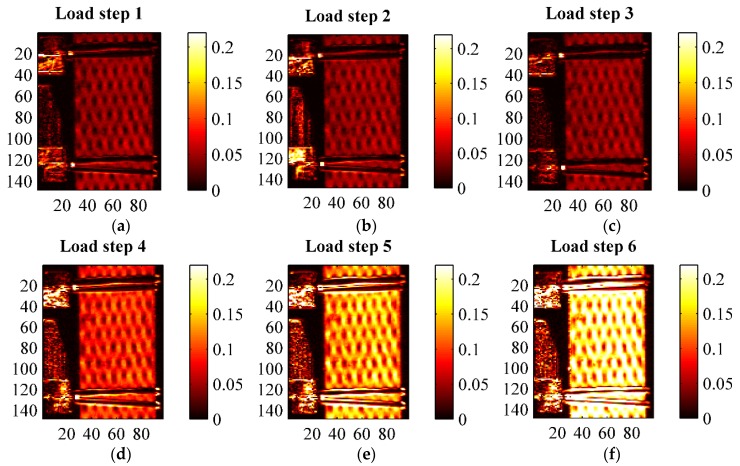
Thermoelastic signal amplitude *ΔT* in (°C) from an HL sample at the various loading steps. (**a**) load step 1; (**b**) load step 2; (**c**) load step 3; (**d**) load step 4; (**e**) load step 5; (**f**) load step 6.

**Figure 13:**
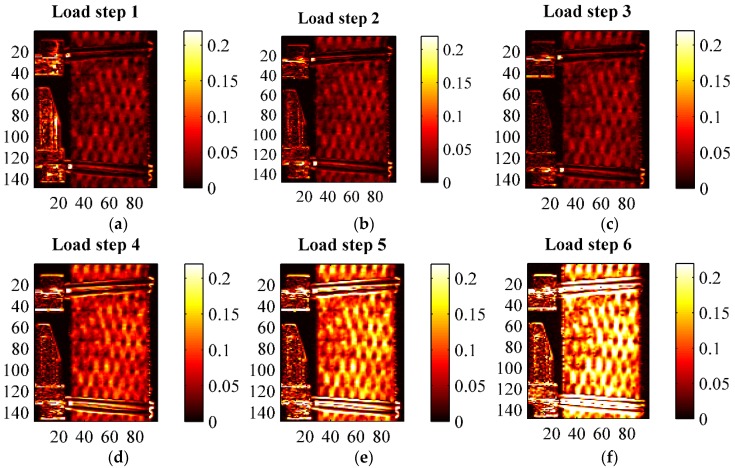
Thermoelastic signal amplitude *ΔT* in (°C) from a VB sample at the various loading steps. (**a**) load step 1; (**b**) load step 2; (**c**) load step 3; (**d**) load step 4; (**e**) load step 5; (**f**) load step 6.

**Figure 14:**
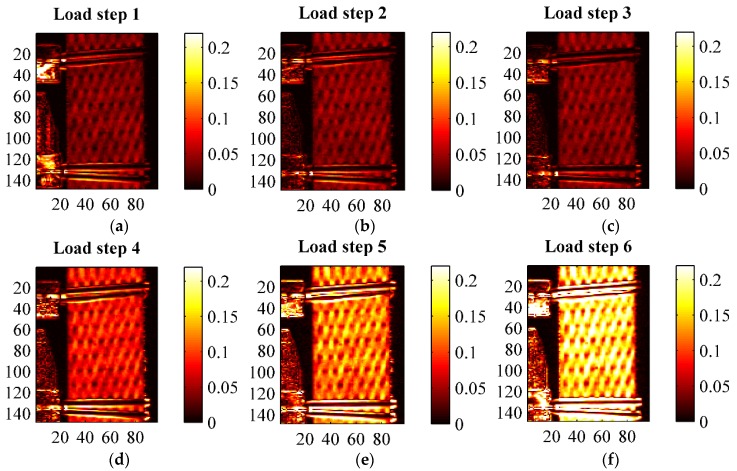
Thermoelastic signal amplitude *ΔT* in (°C) from a RI sample at the various loading steps. (**a**) load step 1; (**b**) load step 2; (**c**) load step 3; (**d**) load step 4; (**e**) load step 5; (**f**) load step 6.

**Figure 15:**
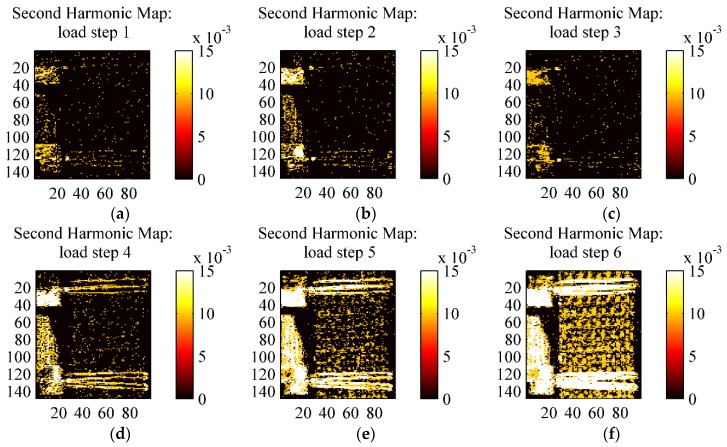
Full field second harmonic signal amplitude in (°C) from an HL sample at the various loading steps. (**a**) load step 1; (**b**) load step 2; (**c**) load step 3; (**d**) load step 4; (**e**) load step 5; (**f**) load step 6.

**Figure 16:**
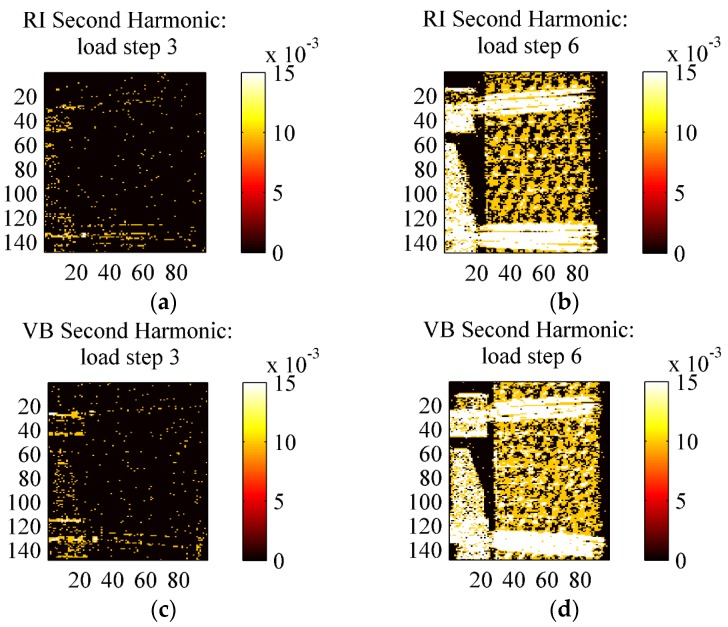
Comparison of second harmonic maps (**a**) RI sample under load step 3; (**b**) RI sample under load step 6; (**c**) VB sample under load step 3; (**d**) VB sample under load step 3.

A second thermographic analysis was carried out consisting of the evaluation of the thermoelastic signal, *i.e.*, *ΔT* of Equation (2), during the fatigue cycling defined in Table 4 and Figure 8. A lock-in filtering was, in particular, implemented to determine the thermoelastic signal, according with the classical approach of Thermoelastic Stress Analysis (TSA) applied to orthotropic materials [26,27]. In particular, the temperature for all cyclic loading steps was acquired under the following constant Thermocamera settings: integration time 1767 μs, frame sub-window 128 × 172 pixels corresponding to a spatial resolution of about 0.23 mm/pixel, instantaneous field of view (iFOV) of 0.25 mm, frame rate 100 Hz, sampling window 30 s. The acquired thermograms were exported from Flir Research IR to Matlab, and further post-processed with an in-house developed algorithm able to obtain both the thermoelastic signal (principal harmonic) and the second harmonic signal by means of a Fast Fourier Transform based lock-in analysis [30]. Results of TSA are reported in Figure 11, Figure 12, Figure 13, Figure 14, Figure 15 and Figure 16. Figure 11 in particular shows an example of amplitude and phase maps of the thermoelastic signal. The acquired sample area is about 15 × 40 mm. It is possible to recognise on the left hand side of the sample the knives of a MTS clip on gage used to monitor strain and fixed on the sample by elastic bands. Figure 12, Figure 13 and Figure 14 collect maps of the thermoelastic signal amplitude from HL, VB and RI at each of the six loading steps applied (see Table 4).

The maps of the thermoelastic signal amplitude in Figure 11, Figure 12, Figure 13 and Figure 14 show a peculiar chessboard like signal pattern, alternating zones of higher and lower amplitude. The higher signal is in particular detected above the zones of the sample surface which expose the weft yarns. This behaviour is also widely discussed in [31] for a GRP composite with similar fabric texture. In such weft tie surfacing zones *Δσ_1_* = *Δσ_T_*, *i.e.*, the orientation of the fibres is such that the transverse Coefficient of Thermal Expansion (CTE) should be considered as the correct coefficient multiplying the axial stress component in Equation (2). It is well known that transverse CTEs for highly anisotropic fibres are much higher (even one order of magnitude) than longitudinal CTEs [27,32]. Hence, the higher thermoelastic signal is mainly due to the higher local CTE. This mismatch in CTEs and the resulting pattern in the thermoelastic signal map also provide a very effective way to verify the quality of fibre alignment and the presence of surface damaged fabric zones. The phase map (see Figure 11b) delivers additional important information about the nature of the acquired thermoelastic signal. In fact, it is possible to observe a constant phase angle and no peculiar pattern such as that of the signal amplitude. This means that adiabatic conditions prevail over the scanned area, which is an essential prerequisite for the effective interpretation of the thermoelastic effect induced temperature change. Although only one phase map is reported in this work, all other phase maps acquired from other samples, and at other load steps, presented similar features.

Figure 12, Figure 13 and Figure 14 show how all materials behaved in a pretty similar way for an equal load step. It is, in particular, reported that the typical features of the thermoelastic signal—pattern, amount of signal and proportionality to the applied load amplitude, phase distribution—were all found also in those load cycles positioned above the knee point. This is considered an important finding. In fact, it states that the behaviour of the cycling material in the CD region is still elastic, since the thermoelastic effect is activated by the reversible volume changes characterising a linear elastic deformation [25]. Even if a complete unloading across the knee region would give rise to inelastic deformations, the materials seem to deploy a local elastic behaviour also in the post knee region. From a comparison of the thermoelastic signal maps, it is found that the signal is similar for load steps 1, 2 and 3, indicating a negligible influence of the mean load (as predicted by first order theories of the thermoelastic effect [25]). As commented in Section 3.3 and shown by Figure 9, the material stiffness is similar during cycle steps 1–3, so an equal load amplitude will result in a similar stress amplitude, and then a similar thermoelastic signal. For load steps 3–6, which have the same average stress but increasing stress amplitude, the thermoelastic signal increases with the stress amplitude, while keeping the same chessboard pattern.

Figure 15 and Figure 16 show the map of the second harmonic signal, *i.e.*, the temperature amplitude carried by the harmonic component at 8 Hz (twice the loading frequency of 4 Hz), obtained by the Fourier Transform Lock-In procedure by selecting a reference signal at 8 Hz [30]. Some works have correlated the presence of dissipation effects to the increase of the second harmonic signal amplitude [33]. This is, for instance, the basis of the Dissipation Mode (D-Mode) analysis proposed by TSA software Altair LI and Thesa by Flir^®^. Figure 15 in particular shows maps of the second harmonic component for all six load steps from an HL sample, while Figure 16 reports the same maps for VB and RI only for loading steps 3 and 5.

Figure 15 in particular shows that the second harmonic signal for the HL material is practically null for loading steps 1–4. A response is starting to develop at load step 5 and consolidates at step 6, even though the amplitude of such signal is much lower than the first harmonic (*i.e.*, the thermoelastic signal), and in the order of 0.01 °C. Figure 16 shows the second harmonic response for the other materials VB and HL, revealing a similar behaviour with HL. This second harmonic response at load step 6 can be regarded as an indication that some dissipative effects start to occur in the material. In this regard, it is interesting to report that the load cycles of step 6 showed a small but easily observed hysteresis area, which was instead absent or negligible during the other lower amplitude cyclic loading steps.

## 5. Conclusions

The present work has investigated the tensile behaviour of quasi-unidirectional Flax Reinforce Epoxy Matrix composites. The investigated materials use the same commercial plain fabric, made of twisted yarn reinforcements, and are fabricated by three traditional lamination processes giving rise to different fibre volume fractions.

The measured tensile behaviour in the reinforcement direction has revealed the typical bilinear behaviour already reported in the literature for similar materials, where the stiffness modulus undergoes a rather marked reduction at low stress/strain values, to then proceed quasi-linearly until the onset of brittle failure.

Repeated loading separated by a recovery period between each test has shown a significantly different behaviour compared to continuous quasi-static loading-unloading cycling. In particular, in the first case, the knee zone seems to change little, and a stiffness hardening is observed, which stabilises after a few loading repetitions. In the second case, a marked inelastic final deformation is obtained at complete unloading, and the stiffness during cycling is higher than that during monotonic loading. In addition, the cycles show a marked hysteresis. It is believed that such different features of the different reloading modes are due to marked intrinsic viscoelastic behaviour of the elementary flax fibres, activated by localised kink band defects as well as by Micro-Fibril-Angle rotations. Higher strain rate cycles lying within the linear portions of the monotonic tensile curve have instead exhibited a local elastic behaviour, with negligible hysteresis and stiffness levels comparable to the initial higher Young’s modulus.

IR thermography has been proposed to monitor the full field temperature evolution during the monotonic and fatigue cycling tensile tests. In the first case, the temperature is initially decreasing linearly due to the onset of the thermoelastic effect. A departure from linearity, associated with the onset of dissipative damage phenomena, has been detected at values of strain well above those of the nonlinear region of the tensile curve. Therefore, whatever produces the non-linear knee region does not seem to have a significant effect in terms of irreversible dissipative heating.

The sampling of temperature during the fatigue cycles has led to the evaluation of the Thermoelastic Signal, according with the procedure developed through Thermoelastic Stress Analysis. The features of the measured thermoelastic signal agree well with the nature of the thermoelastic effect in linear elastic materials. It is then concluded that, even in the post-knee region, and at least for the relatively high strain rates of fatigue cycling (specifically 4 Hz in the present work), the material behaves linearly elastic, at least locally and for load amplitudes not crossing the knee point region. Finally, the derivation of the second harmonic signal has been proposed as a promising approach to detect the load amplitude threshold at which dissipative damage phenomena start to occur.
